# Polyphenols from *Blumea laciniata* Extended the Lifespan and Enhanced Resistance to Stress in *Caenorhabditis elegans* via the Insulin Signaling Pathway

**DOI:** 10.3390/antiox10111744

**Published:** 2021-10-30

**Authors:** Tao Chen, Siyuan Luo, Xiaoju Wang, Yiling Zhou, Yali Dai, Lijun Zhou, Shiling Feng, Ming Yuan, Chunbang Ding

**Affiliations:** College of Life Science, Sichuan Agricultural University, Ya’an 625014, China; chentao293@163.com (T.C.); luosiyuan1998@163.com (S.L.); wxj99537@163.com (X.W.); zhouyiling1997@163.com (Y.Z.); dyl_gdz@163.com (Y.D.); zhoulijun@sicau.edu.cn (L.Z.); fengshilin@outlook.com (S.F.); yuanming@sicau.edu.cn (M.Y.)

**Keywords:** *Blumea laciniata*, antioxidant ability, anti-ageing effect, *Caenorhabditis elegans*

## Abstract

*Blumea laciniata* is widely used as a folk medicine in Asia, but relevant literature on it is rarely reported. We confirmed that polyphenol extract (containing chlorogenic acid, rutin, and luteolin-4-O-glucoside) from *B. laciniata* (EBL) showed strong antioxidant ability in vitro. Hence, in this work, we applied *Caenorhabditis elegans* to further investigate the antioxidant and anti-ageing abilities of EBL in vivo. The results showed that EBL enhanced the survival of *C. elegans* under thermal stress by 12.62% and sharply reduced the reactive oxygen species level as well as the content of malonaldehyde. Moreover, EBL increased the activities of antioxidant enzymes such as catalase and superoxide dismutase. Additionally, EBL promoted DAF-16, a transcription factor, into the nucleus. Besides, EBL extended the lifespan of *C. elegans* by 17.39%, showing an anti-ageing effect. Different mutants indicated that the insulin/IGF-1 signaling pathway participated in the antioxidant and anti-ageing effect of EBL on *C. elegans*.

## 1. Introduction

Ageing and the pursuit of youth have always elicited wide interest from scientific communities [1,2]. In the process of metabolism, free radicals, such as reactive oxygen species (ROS), containing unpaired electrons in a special state are produced to participate in enzymatic or non-enzymatic reactions [3,4]. ROS plays a vital role in signal transduction and the process of different types of metabolism. Normally, there is a delicate balance between the generation and elimination of ROS. However, this equilibrium is easily disturbed by external stimuli. When the balance is broken, the increasing number of ROS will cause more oxidative stress reactions; then, more ROS attack biomacromolecules such as DNA, proteins, and lipids, thus inducing damage to the cellular structure and function, leading to the loss of cell function, gene mutation, aging, diseases, and even cell death [5]. For instance, too high a level of ROS will damage the healthy brain and accelerate the symptoms of Alzheimer’s disease [6]. Meanwhile, more ROS inside the cells will lead to ageing [7]. Some theories have pointed out that antioxidants can increase the antioxidant ability of organisms to defend against oxidative stress, and in some ways they are also able to slow down ageing [8,9,10,11]. Therefore, it is urged to find appropriate antioxidants to reduce oxidation. Hence, natural antioxidants have always been sought after to delay ageing throughout the history of mainkind [11]. Polyphenols from natural plants are always a site of research for finding promising antioxidants, since polyphenols are considered as a potential remedial tool for healthy aging, either as part of a diet or as separate compounds (such as supplements) [12].

Currently, *Caenorhabditis elegans* is a promising model organism to screen natural antioxidant drugs, since the whole genome of the nematode has been sequenced and the homology with human genes reaches more than 40% [13,14,15]. *C. elegans* nowadays is applied to investigate promising herbal medicine for its potential antioxidant ability and, in particular, its anti-ageing effect [8,16]. Extract rich in phenolics, obtained from blueberry, was proven to enhance the resistance of *C. elegans* to stresses such as heat, ultraviolet-B radiation, and paraquat; moreover, it extended the lifespan of *C. elegans* by 22.2~44.4% [17]. Phenolic extract from the leaves of *Caesalpinia mimosoides* enhanced oxidative stress resistance and prolonged the lifespan of *Caenorhabditis elegans* through the DAF-16/FOXO pathway [18]. Olive polyphenols increased the healthspan of *C. elegans*, and catechinic acid, a natural polyphenol compound, extended the lifespan of *Caenorhabditis elegans* via mitophagy pathways. [19,20]. Dietary antioxidants such as vitamins and plant-based metabolites, such as polyphenols (resveratrol, anthocyanins, flavanones, and isoflavones, in particular), slow ageing by decreasing ROS generation in cells, thus improving the lifespan of living organisms [21].

*Blumea laciniata* is a widely used herbal folk medicine in Asia. In our previous work, the antioxidant capacity of *B. laciniata* was correlatively associated with the content of polyphenols. Additionally, polyphenol extract from *B. laciniata* (EBL) excellently scavenged free radicals and protected HeLa cells against oxidative stress induced by H_2_O_2_, showing strong antioxidant ability in vitro [22]. Though EBL showed strong antioxidant ability in vitro, the inner environment is complex, and whether EBL still exerts an excellent antioxidant capacity there is unknown. Therefore, in this present work, we applied *C. elegans* to further evaluate the antioxidant potential and anti-ageing effect of EBL in vivo.

## 2. Materials and Methods

### 2.1. Materials and Chemicals

*Blumea laciniata* was obtained from Zigong, Sichuan Province, China, at 29°18′41″ N, 104°47′31″ E. The plant was identified by professor Chao Hu, Department of Botany, College of Life Sciences, Sichuan Agricultural University. The whole plant was washed with distilled water 3 times and dried at 45 °C for 4 days. Then, the whole herb, including the underground part, was ground into a fine powder.

Peptone, tryptone, and yeast extract were purchased from OXOID Co., Ltd. (Besting Stoke, UK). Ethanol, sodium chloride, magnesium sulfate, calcium chloride, and others were obtained from Chengdu Kelong Chemistry Co., Ltd. (Chengdu, China). A reactive oxygen species (ROS) kit, superoxide dismutase kit (SOD), catalase kit (CAT), malonaldehyde kit (MDA), and total antioxidant capacity kit (T-AOC) were all bought from the Nanjing Jiancheng Bioengineering Institute (Nanjing, China).

### 2.2. The Preparation of Polyphenol Extract from Blumea laciniata (EBL)

The powder of *Blumea laciniata* was mixed with 80% ethanol for 45 min at 40 °C with the assistance of ultrasound. Then, the solution was concentrated by rotary evaporation and lyophilized to obtain polyphenol extract from *Blumea laciniata* (EBL). The content of polyphenols in EBL was 45~48%, and the chemical compositions were assessed by high-performance liquid chromatography, as shown in our previous work [22]. Five phenolic components were identified as chlorogenic acid, isoorientin, rutin, luteolin-4-O-glucoside, and cinnamic acid.

### 2.3. C. elegans Strains and Maintenance

Wild-type Bristol N2, transgenic nematode strains, and *Escherichia coli* OP50 were kindly provided by the *Caenorhabditis* Genetics Center. The transgenic worms applied in this work were TJ356 *daf-16*::GFP (*zls356*), CF1038 *daf-16*(*mu86*)*I*., CB1370 *daf-2* (*e1370*)*III*., TJ1052 *age-1* (*hx546*)*II*., VC204 *akt-2*(*ok393*)*X*., VC345 *sgk-1*(*ok538*)*X*., and EU1 *skn-1*(*zu67*)*IV*. All nematodes were maintained on a nematode growth medium (NGM) plate with a layer of OP50 as food at 20 °C.

EBL was dissolved in dimethyl sulfoxide (DMSO) and poured onto an NGM plate when the medium was 50 °C during the liquid stage (the final concentration of DMSO was less than 0.1%). The positive control for thermal stress was ascorbic acid (Vc), at a concentration of 10 μg/mL.

### 2.4. The Food Clearance Assay

The food clearance assay was carried out according to the previous work [23]. Synchronized worms were obtained by the sodium hypochlorite method [24]. L4 larvae were placed in a 96-well plate with EBL and 5-fluorouracil (35 mM). The absorbance of the plate was read at 600 nm every day.

### 2.5. Fertility Assay

Pregnant worms were placed on the nematode growth medium (NGM) for 2 h to lay eggs, and were incubated at 20 °C to obtain L1 worms. Then, the worms were transferred to a new plate with the presence or absence of EBL. When the worms began laying eggs, the worms were put on a new NGM plate until the worms stopped laying. Additionally, the eggs were incubated at 20 °C for 24 h. Then, the hatched worms were recorded. Ten worms were utilized for each concentration.

### 2.6. Thermal Stress Assay

Synchronized L1 worms were placed on the plate for 48 h of treatment. Then, the worms were shifted to 35 °C for 5 h of exposure. Subsequently, the worms were placed at 20 °C for 24 h, and the living worms were counted. Thirty worms were used for each independent experiment.

### 2.7. Assessment of Lifespan

Synchronized L1 worms were put on an NGM plate containing EBL, or not, at 20 °C. During the pawning period, the worms were transferred onto a new plate every day (60 worms per plate). When the worms stopped laying, the nematodes were transferred onto a new NGM every other day. The numbers of dead worms were counted and the living worms were observed.

### 2.8. Assessment of the Level of ROS

Synchronized L1 worms were put on an NGM plate containing EBL or not at 20 °C for 48 h. Then, for stress conditions, the worms were placed at 35 °C for 1.5 h; for normal conditions, the worms were placed at 20 °C for 1.5 h. Then, the worms were collected and washed with K medium 5 times to remove OP50. An ROS kit was used to stain the ROS in *C. elegans* according to the manufacturer’s instructions. The fluorescence intensity of the ROS was read at the excitation wavelength of 488 nm and the emission wavelength of 525 nm. Moreover, the worms were put on a glass slide to observe the level of ROS under a fluorescence microscope (OLYMPUS BX53).

### 2.9. Assessment of the Activities of Antioxidant Enzymes and the Content of MDA

Synchronized L1 worms were placed on an NGM plate with and without EBL for 48 h at 20 °C. Then, for thermal stress, the worms were shifted to 35 °C for 1.5 h; for normal conditions, the worms were placed at 20 °C for 1.5 h. Subsequently, the worms were washed with a K medium 5 times to remove OP50. Additionally, all worms were frozen at −80 °C for 10 min and then crushed. Then, the supernatant was centrifuged at 12,000× *g* rpm/min for 10 min to obtain the protein solution. The solution was added into a new tube for subsequent assays. The content of protein was determined by the previous method [25], and the activities of CAT, SOD, and T-AOC, as well as the content of MDA, were measured according to the kit instructions.

### 2.10. Visualization of the DAF-16 Transcription Factor

The transgenic strain TJ356 carried a GFP on the DAF-16 transcription factor, thus making it easy to observe the localization in *C. elegans*. Synchronized L1 larvae were exposed to EBL for 48 h; the worms were anesthetized by sodium azide (35 mM) and the localization of the DAF-16 transcription factor was observed under a fluorescence microscope (OLYMPUS BX53). There were 10 worms in each independent experiment.

### 2.11. Statistics

All data obtained in this work were analyzed using GraphPad Prism 8.0. Comparisons of lifespan curves were made by log-rank tests. Analyses of quantitative data were assessed with one-way ANOVA. Additionally, an unpaired, two-tailed t test was carried out to evaluate statistical differences in mean values. All data were shown as mean ± SD. *p* < 0.05 was considered to be significant.

## 3. Results

### 3.1. EBL Showed No Toxicity on C. elegans

**Figure 1:**
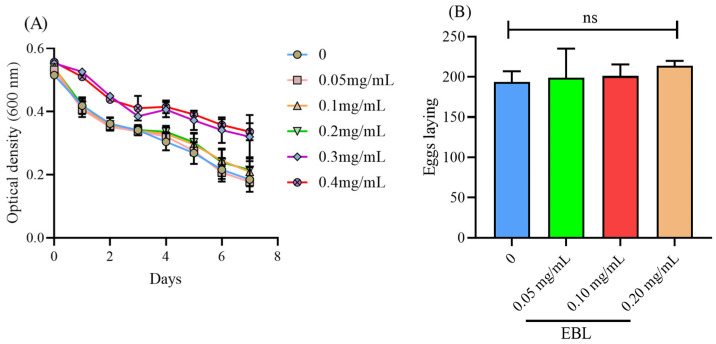
The effect of EBL on (**A**) food intake ability and (**B**) the brood side. Notes: ns meant not significant.

A high concentration of drugs may have negative effects on *C. elegans*. Therefore, a suitable concentration of EBL to use on *C. elegans* shall be determined. As shown in Figure 1A, 0.3 and 0.4 mg/mL of EBL slowed down food intake ability, while 0–0.2 mg/mL showed no evident impact on *C. elegans*. Hence, three concentrations (0.05, 0.10, and 0.20 mg/mL) were adopted to evaluate the effect on reproduction ability. As shown in Figure 1B, the worms in the blank group laid 193.6 ± 13.46 eggs. Additionally, after treatment with EBL, the numbers of laid eggs were 198.9 ± 36.20, 201.2 ± 14.39, and 213.8 ± 6.150, respectively, showing no significant difference compared with the blank group (*p* > 0.05). Hereby, 0.05, 0.1, and 0.2 mg/mL of EBL were selected for subsequent assays.

### 3.2. EBL Enhanced the Survival of C. elegans under Thermal Stress

**Figure 2:**
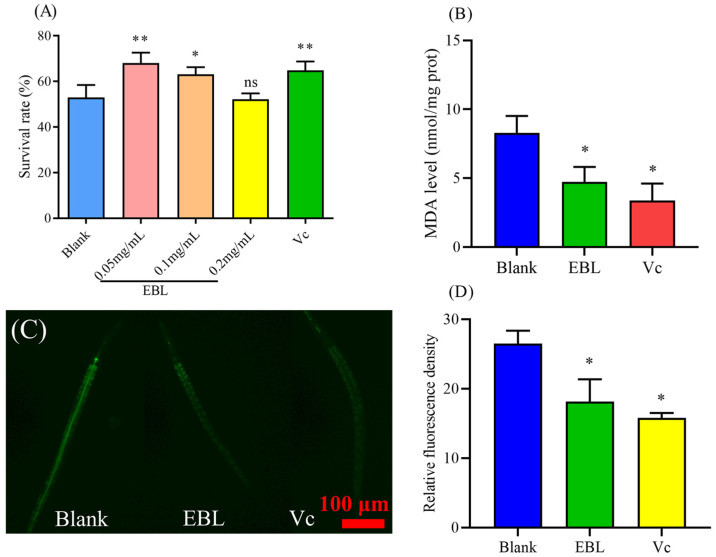
The impact of EBL on (**A**) the survival rate of *C. elegans*, (**B**) the content of MDA, (**C**) the level of ROS under thermal stress, and (**D**) the relative fluorescence density of ROS in *C. elegans*. The concentration of EBL for the determination of MDA and ROS was 0.1 mg/mL, considering the results of lifespan assays where only 0.1 mg/mL of EBL extended the lifespan of *C. elegans*. Vc meant ascorbic acid (10 μg/mL). Notes: * and ** meant *p* < 0.05 and *p* < 0.01, respectively. Additionally, ns meant not significant.

Normally, *C. elegans* lives at 20 °C. When the worms were shifted to a higher temperature they were stressed, the level of ROS will increase, and the survival of *C. elegans* under thermal stress will decrease [26]. As shown in Figure 2A, the survival in the blank group was 52.94 ± 5.48%, and with 48 h of treatment of 0.05 and 0.1 mg/mL of EBL the survival rates were significantly enhanced by 17.63% and 12.62%, respectively. However, 0.2 mg/mL of EBL failed to prolong survival (*p* > 0.05). Therefore, 0.05 and 0.1 mg/mL of EBL enhanced the resistance of *C. elegans* against thermal stress, showing obvious protection.

### 3.3. EBL Reduced the Content of ROS and MDA in C. elegans under Thermal Stress

There is a close correlation between stress resistance and antioxidant ability [27]. Under thermal stress, the increasing level of ROS will attack biomacromolecules in cells, such as DNA and proteins [28]. Natural plant extract then enhances the antioxidant defense system in *C. elegans*, thus eliminating ROS and improving the resistance of *C. elegans* to stresses, such as thermal stress and oxidative stress [29,30]. Hence, we determined the effect of EBL on the level of ROS in *C. elegans*. As shown in Figure 2C, the level of ROS was different in different groups. The level of ROS in the worms pretreated with EBL was significantly decreased, by 31.60% (Figure 2D), meaning the strong resistance ability in *C. elegans* treated with EBL was associated with scavenging on ROS.

As a biomarker, MDA can reflect the degree of free radical attack and lipid peroxidation; therefore, the content of MDA is considered as the main symbol for oxidative damage in the process of aging and metabolism [31]. As shown in Figure 2B, the content of MDA in the blank group was substantially high. Additionally, with the treatment of EBL, the level of MDA in worms was sharply decreased by 41.46%. This result indicated that EBL reduced lipid peroxidation and relieved the oxidative damage induced by thermal stress.

### 3.4. EBL Increased the Activities of Antioxidant Enzymes in C. elegans under Thermal Stress

**Figure 3:**
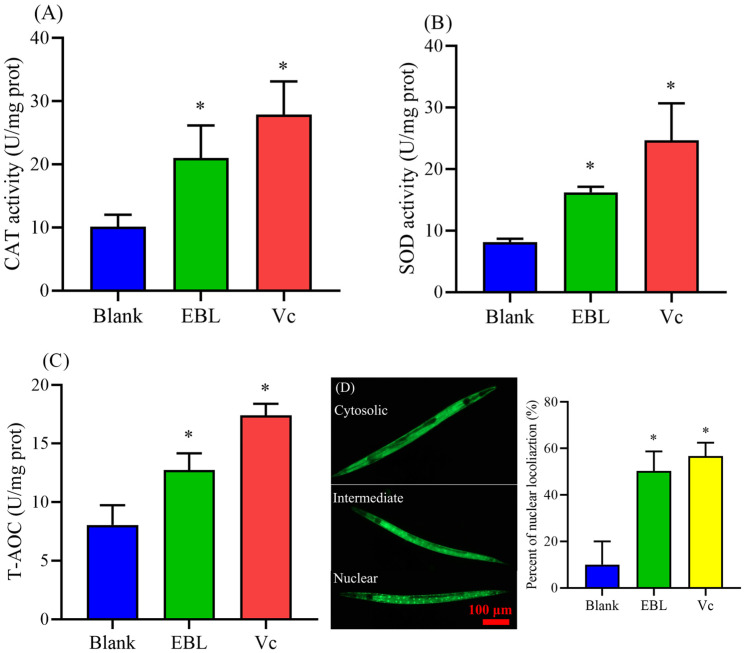
The effect of EBL on the activities of antioxidant enzymes (**A**) CAT and (**B**) SOD, and the (**C**) total antioxidant capacity (T-AOC) in *C. elegans* under thermal stress. (**D**) After treatment with EBL for 48 h, the DAF-16 transcription factor was localized to the nucleus in *C. elegans*. The concentration of EBL was 0.1 mg/mL. Vc meant ascorbic acid (10 μg/mL). Note: * meant *p* < 0.05.

The antioxidant defense system plays an important role against the damage induced by oxidation [32]. After treatment with EBL, the degree of oxidative damage was decreased, which might be related to the regulation of the antioxidant defense system. Therefore, we further evaluated the activities of antioxidant enzymes. As shown in Figure 3A, the activity of CAT was significantly increased by 46.73% compared with the blank group. Additionally, the activity of SOD was also enhanced by 43.84% (Figure 3B). The activities of the two main antioxidant enzymes in *C. elegans* were both improved, thus making the total antioxidant ability stronger (Figure 3C). Therefore, EBL activated the antioxidant defense system; the antioxidant enzymes then eliminated the increasing ROS, improving survival under heat stress, showing excellent antioxidant ability in vivo.

### 3.5. EBL Promoted the Localization of DAF-16 to the Nucleus

To further explore whether the effect of EBL on *C. elegans* was associated with *daf-16*, a main regulatory center of metabolism, stress response, and life cycle [33,34], a TJ356 transgenic worm was applied to observe the location of the DAF-16 transcription factor in *C. elegans*. The genes of antioxidant enzymes are located downstream of *daf-16*. As shown in Figure 3D, after 48 h of treatment with EBL, the frequency of the DAF-16 transcription factor being located in the nucleus was increased significantly by 40.30%, meaning EBL activated DAF-16.

### 3.6. EBL Failed to Increase the Survival of Mutants

**Figure 4:**
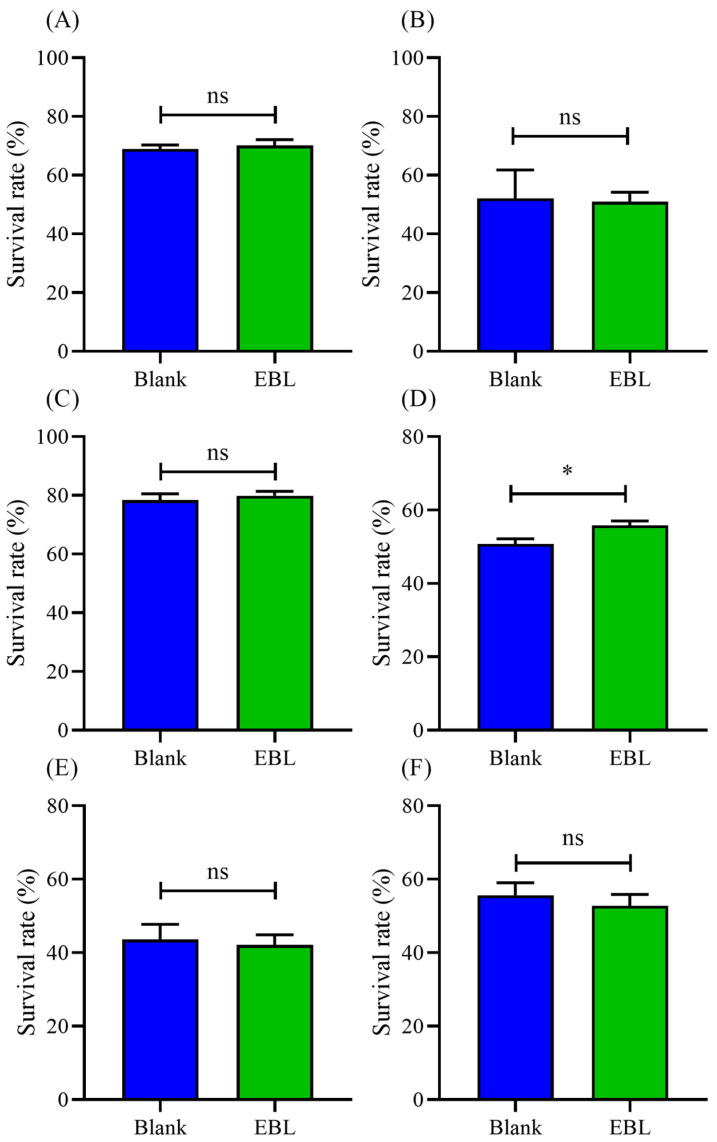
The survival of different mutants under thermal stress. (**A**) TJ1052 *age-1* (*hx546*), (**B**) CF1038 *daf-16*(*mu86*), (**C**) CB1370 *daf-2* (*e1370*), (**D**) EU1 *skn-1*(*zu67*), (**E**) VC204 *akt-2*(*ok393*), and (**F**) VC345 *sgk-1*(*ok538*). The concentration of EBL was 0.1 mg/mL. Note: * meant *p* < 0.05 and ns meant not significant.

EBL improved the survival of wild-type *C. elegans* under thermal stress (Figure 2A). Hence, we used six types of mutants to evaluate the mechanism. As shown in Figure 4A, the survival of the TJ1052 strain, missing the *age-1* gene, was not increased after being treated with EBL, meaning *age-1* was a key gene in the process of increasing resistance capacity, induced by EBL. Similarly, EBL failed to enhance the survival of CF1038 (Figure 4B), CB1370 (Figure 4C), VC204 (Figure 4E), and VC345 (Figure 4F). Therefore, *daf-16*, *daf-2*, *akt-2*, and *sgk-1*, four genes, were necessary for EBL to strengthen the resistance of *C. elegans* under thermal stress. Besides, EBL significantly increased the survival of EU1 nematodes by 5.01% (Figure 4D). However, EBL enhanced the survival rate of wild-type worms by 12.62%, which was signficiantly different from the effect had on the EU1 strain. Hence, EBL could not fully restore resistance in EU1 worms, suggesting skn-1 was partly participating in the process of EBL.

### 3.7. EBL Extended the Lifespan of C. elegans

**Figure 5:**
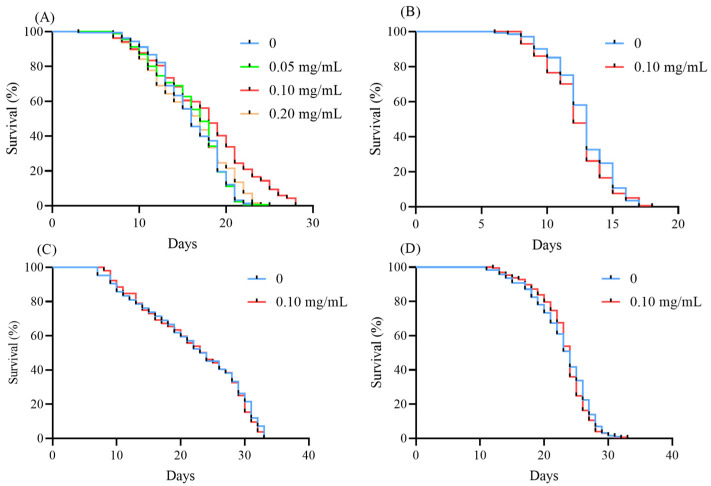
The effect of EBL on the lifespans of (**A**) wild-type worms. Additionally, EBL showed an extended effect on N2 worms. The effect of EBL on the mutants involving the insulin signaling pathway, such as (**B**) TJ1052 *age-1* (*hx546*), (**C**) CF1038 *daf-16*(*mu86*), and (**D**) CB1370 *daf-2* (*e1370*) strains.

There is a close connection between antioxidant ability and anti-ageing capacity [35]. Since EBL showed strong antioxidant ability, we wondered whether EBL also exhibited a positive effect on the lifespan of *C. elegans*. As shown in Figure 5A, 0.10 mg/mL of EBL extended the lifespan of *C. elegans*. In the blank group, the worms lived 16.33 ± 0.58 days, while after treatment with EBL the worms had a mean lifespan of 19.17 ± 1.61 days, which was 17.39% longer than that of the blank group. However, 0.05 and 0.20 mg/mL of EBL failed to prolong the lifespan of *C. elegans* (Table 1).

### 3.8. EBL Failed to Extend the Lifespan of Mutants

To further explore the mechanism, three mutants were applied to evaluate the anti-ageing effect of EBL. As shown in Figure 5B, the lifespans of TJ1052 between the blank group and the EBL group were not significantly changed (*p* > 0.05). As the results show in Figure 5C, the mean lifespan of CF1038 was 12.67 ± 0.58 days, and with the treatment of EBL the survival time was not prolonged (*p* > 0.05). Additionally, EBL did not extend the lifespan of CB1370 worms (Figure 5D). Hence, *age-1* and *daf-2* were necessary for EBL to extend the lifespan of *C. elegans*. *daf-16* was located downstream of *age-1* and *daf-2*. We used the CF1038 strain to assess the role of *daf-16* in the regulation of the longevity of EBL. Therefore, the three genes of *daf-2*, *age-1*, and *daf-16* were essential for EBL to exert an excellent anti-ageing effect.

### 3.9. EBL Kept a Normal Level of ROS and MDA in C. elegans under Normal Conditions

**Figure 6:**
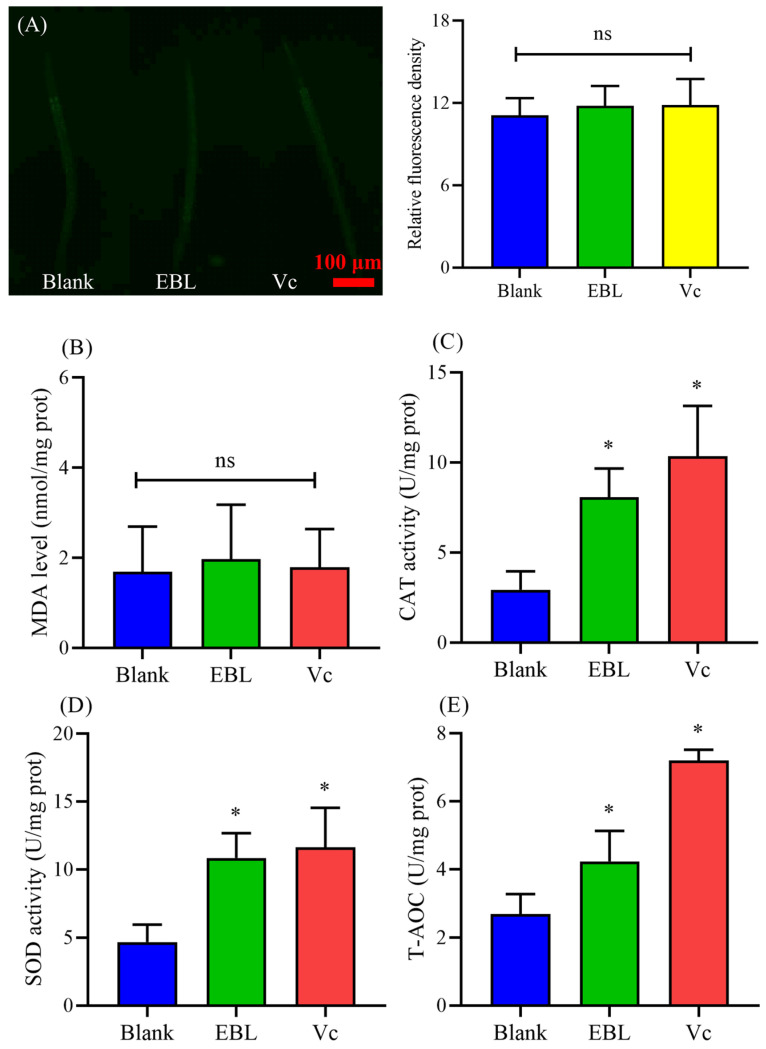
The effect of EBL on the antioxidant defense system of *C. elegans* under normal stress. (**A**) The level of ROS and (**B**) the content of MDA. The activities of (**C**) CAT and (**D**) SOD enzymes. (**E**) The total antioxidant capacity (T-AOC). The concentration of EBL was 0.1 mg/mL. Vc meant ascorbic acid (10 μg/mL). **Note:** * meant *p* < 0.05 and ns meant not significant.

Since EBL possessed strong antioxidant ability in vivo, we assumed that EBL might regulate the antioxidant defense system to extend the lifespan of *C. elegans*. Hence, we determined the level of ROS and the content of MDA under normal stress. As shown in Figure 6A, the level of ROS was not significantly changed. Meanwhile, the content of MDA was also not changed (Figure 6B). These results indicate that EBL showed no stimulatory effect on *C. elegans* and also kept the worms at a normal physiological level.

### 3.10. EBL Increased the Antioxidant Ability of C. elegans under Normal Conditions

To further verify our findings, the antioxidant ability of *C. elegans* was evaluated under normal stress. As shown in Figure 6C, the activity of CAT was greatly increased by 175.71%. Additionally, after being treated with EBL, the activity of SOD was also significantly improved by 131.57% (Figure 6D). These two enzymes were the main defenders in the antioxidant defense system. Thus, the total antioxidant capacity of *C. elegans* was enhanced by 57.46% (Figure 6E). Therefore, EBL activated the antioxidant defense system in *C. elegans* to maintain normal levels of ROS and MDA, leading to a long life.

## 4. Discussion

EBL was proven to protect HeLa cells against oxidative damage induced by H_2_O_2_, similarly to other phenols extract from plants [22]. However, inner environments are substantially complex, and it is difficult for cell assays to reflect the regulation between complex environments in the body. Therefore, in this work, we used *C. elegans* to comprehensively evaluate the antioxidant ability of EBL in vivo. Under heat stress, more ROS were generated and they attacked the body, leading to a low survival rate [36]. As eventually shown by the results (Figure 2A), EBL increased the survival of *C. elegans* under thermal stress, indicating that EBL exerts a certain antioxidant capacity on *C. elegans*. In the body, the antioxidant defense system is the main defender, and antioxidant enzymes such as CAT and SOD in particular are the principal agents used to eliminate increasing numbers of free radicals [37]. EBL promoted the activities of CAT and SOD under thermal stress. Thus, the total antioxidant capacity of *C. elegans* was enhanced (Figure 3A–C). Moreover, as the antioxidant enzymes were stimulated, the level of ROS was sharply reduced, and the oxidative damage in addition to lipid peroxidation were speculated to be reduced as the content of MDA was decreased. This process is commonly observed among other natural products acting in *C. elegans*. Phenolic extract from olive leaves was proven to activate the activities of CAT, SOD, and glutathione peroxidase (GSH-Px), which then reduce the level of ROS and enhance the resistance of *C. elegans* to thermal stress [23]. Polyphenolic extract from *Anacardium occidentale* stimulated the expression of stress response genes such as SOD-3 and GST-4, which then reduced the level of ROS and increased the survival of *C. elegans* under stress conditions [38]. Therefore, we speculated that EBL activated the antioxidant defense system of *C. elegans* against harsh environments, such as the increasing level of ROS, thereby protecting worms.

The insulin/IGF-1 signaling pathway (IIS) is one of the most important pathways to regulate aging, development, and stress. The DAF-16 transcription factor, a homologue to FOXO in higher animals is a key factor in the IIS pathway, and the expression of *daf-16* is closely associated with the resistance of *C. elegans* to heat, oxidation, and metals [33,34]. Hence, we wondered whether EBL regulated the IIS pathway to strengthen the resistance of *C. elegans* to thermal conditions. Subsequently, the effect of EBL on the location of the DAF-16 transcription factor was evaluated, and the frequency with which the DAF-16 transcription factor was located in the nucleus was more than that treated without EBL (Figure 3D). Thus, this outcome supported our hypothesis. As with other naturally phenolic extracts, *Agrimonia procera* Wallr. extract increased the resistance of *C. elegans* against thermal stress by promoting DAF-16 into the nucleus [39]. Meanwhile, the genes of antioxidant enzymes are located downstream of *daf-16*. The DAF-16 transcription factor is stimulated into the nucleus, the expression of *daf-16* gene is activated, and the expression of downstream genes such as *sod-3* and *ctl-1* is then promoted, which is shown to increase the activities of antioxidant enzymes [33,34]. However, the IIS pathway is a complex signal transduction pathway. The activity of *daf-16* is regulated by the upstream *age-1* and *daf-2* genes, as well as by the phosphorylation of AKT-1, AKT-2, SGK-1, JNK-1, and CST-1 [40]. Upon further exploration, the survival of mutants such as TJ1052 *age-1* (*hx546*), CF1038 *daf-16*(*mu86*), CB1370 *daf-2* (*e1370*), VC204 *akt-2*(*ok393*), and VC345 *sgk-1*(*ok538*) were not significantly changed, meaning that the IIS pathway was involved in enhancing the resistance induced by EBL. Therefore, in a word, after being treated with EBL, the phenomonon that the activities of CAT and SOD were significantly enhanced was associated with the regulation of the IIS pathway and more of the DAF-16 transcription factor in the nucleus, induced by EBL, thus reducing the high level of ROS in worms, exhibiting an excellent antioxidant capacity in vivo.

Another strain, EU1 *skn-1*(*zu67*), is not included in the IIS pathway, which is also a vital gene that regulates the resistance to stress. SKN-1 functions in the p38 MAPK pathway to regulate the oxidative stress response, and in parallel to DAF-16/FOXO in the DAF-2-mediated insulin/IGF-1-like signaling pathway to regulate adult lifespan [41]. Additionally, other research has indicated that SKN-1 can be directly phosphorylated by the AKT-1, AKT-2, and SGK-1 kinases that lie downstream of DAF-2 in the insulin signaling pathway [40,42]. Therefore, we assessed the participation of *skn-1*. EBL increased the survival of the EU1 strain by 5.01%, which was relatively lower than the effect on wild-type *C. elegans* (12.62%). This result indicated that *skn-1* gene partly took part in the process of increasing the resistance of *C. elegans*. In previous research, other natural plants also regulated the IIS pathway to regulate the resistance of *C. elegans*. Chlorogenic acid protected *C. elegans* against thermal stress via HIF-1, HSF-1, and autophagy [43]. Phenolic extract from *Anacardium occidentale* attenuated intracellular reactive oxygen species (ROS) via the DAF-16/FoxO and SKN-1/Nrf-2 signaling pathways [38]. A Chinese traditional herbal tea, containing eighteen phenolic compositions, enhanced stress resistance (to oxidative stress and heat stress) in *C. elegans*, and this protective mechanism was positively correlated with the insulin/insulin-like growth factor signaling (IIS-)-dependent manner [44]. All in all, EBL increased the resistance of *C. elegans* to thermal stress via the IIS pathway, accompanying other pathways, such as *skn-1*, as well as other natural phenols from plants.

Usually, antioxidant ability is associated with an anti-ageing effect [45,46,47]. Polyphenol extract from *Rosa rugosa* tea enhanced the thermotolerance and resistance to oxidative stress, in addition to prolonging the lifespan, of *C. elegans* [48]. Grape pomace extracts also increased the survival of *C. elegans* under stress conditions and extended its lifespan [36]. Since EBL showed a strong antioxidant ability in vitro and in vivo, we wondered whether EBL could prolong the lifespan of *C. elegans*. As eventually seen in the results, EBL significantly extended the mean lifespan of *C. elegans* by 17.31% compared with the blank group, showing excellent anti-ageing ability. Therefore, we further explored the antioxidant ability of *C. elegans* under normal stress to find the link between antioxidant potential and anti-ageing ability. ROS and MDA, as the main markers for aging, were not significantly changed. As the results showed, the activities of CAT and SOD were surprisingly promoted. The total antioxidant capacity of *C. elegans* was also enhanced (Figure 6C–E). Hence, we assumed that EBL activated the antioxidant defense system to keep *C. elegans* at a normal physiological level; thus, the increasing level of ROS could be timely scavenged, avoiding oxidative damage. The life cycle of *C. elegans* is controlled by several main pathways, especially the IIS pathway. Our findings further demonstrated that *age-1*, *daf-16*, and *daf-2* were necessary for EBL to exhibit the longevity effect, meaning that the IIS pathway participated in the process. In the IIS pathway, *daf-2* and *age-1* genes inhibit the expression of *daf-16*, which is the main factor that regulates the reproduction and life cycle of *C. elegans*. Since EBL activated the transcription of DAF-16 into the nucleus, we assumed that EBL might suppress the IIS pathway to promote the lifespan of *C. elegans*. The chemical compositions of EBL were identified previously [22]. Chlorogenic acid was a main component. Moreover, chlorogenic acid was proven to exhibit an effect on the ageing of *C. elegans*, mainly via DAF-16 in the insulin/IGF-1 signaling pathway [49]. A natural polyphenol compound, catechinic acid extended the lifespan of *Caenorhabditis elegans* via mitophagy pathways [20]. Therefore, in this work, EBL exhibited excellent antioxidant ability and anti-ageing effect through the IIS pathway.

Normally, the main damage induced by metals and other hazardous substance is acute or chronic oxidative damage to the organ [50]. Disruption of metal ion homeostasis may cause oxidative stress, increasing the level of ROS and subsequently inducing DNA damage, lipid peroxidation, protein modification, and other effects, including cancer, cardiovascular disease, diabetes, and others [51]. Similarly, resveratrol attenuated iron-induced toxicity in *C. elegans* [52]. A traditional Chinese medicine, *Tetrastigma hemsleyanum*, protected *C. elegans* from the toxicity induced by acrylamide through reducing the level of ROS [53]. Hence, we suspected that EBL, possessing strong antioxidant ability, could protect the organ from oxidative damage, which would be a promising perspective.

## 5. Conclusions

In this work, we used *C. elegans* to further evaluate the antioxidant ability of EBL in vivo. As eventually shown by the results, EBL promoted more of the DAF-16 transcription factor into the nucleus and enhanced the activities of antioxidant enzymes such as SOD and CAT. Then, the inner level of ROS and the content of MDA were sharply reduced, increasing the survival rate under oxidative stress conditions. Meanwhile, EBL also prolonged the lifespan of *C. elegans* by increasing the antioxidant capacity via the IIS pathway. Besides, EBL failed to extend the lifespan and survival rate of mutants such as TJ1052, CF1038, and CB1370, meaning that EBL might regulate the insulin signaling pathway to enhance the health of *C. elegans*. These results indicate EBL possesses strong antioxidant ability in vivo and that EBL may be a promising potential agent for further anti-ageing pharmacological research.

## Figures and Tables

**Table 1:** The mean lifespans of four types of worms in the presence of EBL.

Stains	Treatment	Numbers of Trials	Mean Lifespan (Days)	Significance
Wild-type	-	158	16.33 ± 0.58	
	0.05 mg/mL of EBL	161	17.00 ± 1.00	ns
	0.10 mg/mL of EBL	139	19.17 ± 1.61	*
	0.20 mg/mL of EBL	126	16.50 ± 0.87	ns
TJ1052 *age-1* (*hx546*)	-	84	23.33 ± 1.16	
	0.10 mg/mL of EBL	104	23.33 ± 0.76	ns
CF1038 *daf-16*(*mu86*)	-	141	12.67 ± 0.58	
	0.10 mg/mL of EBL	157	12.67 ± 0.43	ns
CB1370 *daf-2* (*e1370*)	-	187	23.33 ± 1.15	
	0.10 mg/mL of EBL	197	23.67 ± 0.58	ns

**Notes:** ns meant not significant and * meant *p* < 0.05.

## Data Availability

Data is contained within the article.
